# Association between endometriosis and gut microbiota: systematic review and meta-analysis

**DOI:** 10.3389/fmicb.2025.1552134

**Published:** 2025-05-07

**Authors:** Li Yuanyue, Ouyang Dimei, Liu Ling, Ren Dongyan, Wu Xiaomei

**Affiliations:** ^1^Department of Gynecology, The First People’s Hospital of Yunnan Province, Affiliated Hospital of Kunming University of Science and Technology, Kunming, Yunnan, China; ^2^Yunnan Provincial Key Laboratory of Clinical Virology, Kunming, Yunnan, China; ^3^Department of Information, The First People’s Hospital of Yunnan Province, Kunming, Yunnan, China

**Keywords:** endometriosis, gut microbiota, dysbiosis, systematic review, meta-analysis

## Abstract

**Background:**

Endometriosis, a complex gynecological disorder, has been increasingly linked to gut microbiota dysbiosis, suggesting its potential role in disease pathogenesis.

**Methods:**

This systematic review and meta-analysis explore the association between gut microbiota and endometriosis by evaluating alpha and beta diversity measures across 11 studies involving 1,727 women, including 433 diagnosed with endometriosis and 1,294 controls. Statistical analysis was performed utilizing either random effects models or fixed models by Revmen5.2 and STATA softwares.

**Results:**

Significant differences in alpha diversity between endometriosis and control groups were observed using the Shannon Index (SMD = 0.39; *p* < 0.00001), Subgroup analysis showed significant differences for Chinese (SMD = 0.48; 95% CI = 0.14 to 0.82; *p* = 0.006; *I*^2^ = 30%), Swedish, (SMD = 0.55; 95% CI = 0.27 to 0.83; *p* = 0.0001; *I*^2^ = 30%) and Spanish (SMD = 0.34; 95% CI = −0.02 to 0.85; *p* < 0.06; *I*^2^ = 27%), compared to others which highlighting the correlation between gut microbiota diversity and endometriosis across different demographic groups. The Simpson Index also revealed a notable difference in richness (SMD = 0.91; *p* = 0.03). However, no significant differences were detected using the Chao Index (SMD = 0.37; *p* = 0.11). These findings underscore the importance of diversity measures in understanding gut microbiota’s role in endometriosis. Seven studies employed PCoA, two used the Bray–Curtis dissimilarity index, and one performed PCA, revealing notable dissimilarities in gut microbiota composition between the groups. Using the Newcastle-Ottawa Scale, most studies scored ≥7 stars, indicating high quality. The funnel plot and Egger’s linear regression analysis indicated no publication bias.

**Conclusion:**

This study highlights significant alterations in gut microbiota diversity and composition in women with endometriosis, emphasizing the potential role of gut microbiota in its pathogenesis. Future research should focus on standardizing reporting methods to facilitate deeper quantitative analyses.

**Systematic Review Registration:**

PROSPERO (registration number RD42024611701).

## Introduction

Endometriosis is a gynecological disorder affecting approximately 10% of women globally (Lamceva et al., 2023), characterized by its chronic, inflammatory, and estrogen-dependent nature. Individuals with this condition often experience symptoms like dysmenorrhea, chronic pelvic pain, infertility, urinary issues, and gastrointestinal disturbances (Zondervan et al., 2018). Mental health issues, including depression and anxiety (Laganà et al., 2017), are also common. The disease involves the ectopic growth of endometrial tissue outside the uterine cavity, typically on the ovaries and peritoneal surfaces. The most widely recognized theory for its pathogenesis is retrograde menstruation, which proposes that menstrual blood containing endometrial cells refluxes into the pelvic cavity, where these cells implant on pelvic organs. Over time, this causes fibrotic lesions, vascularization, and nerve fiber regeneration (Ja, 1927). However, this theory does not fully explain the discrepancy between the high likelihood of menstrual reflux and the lower incidence of endometriosis.

Recent studies have provided strong evidence that endometriosis is a multifactorial disease, driven by complex interactions between genetic, immune, and endocrine factors that contribute to the development and progression of ectopic lesions (Lamceva et al., 2023). In recent years, the role of microbiota dysbiosis in various diseases, particularly endometriosis, has gained significant attention. Microbiota play a crucial role in maintaining human health by protecting against pathogenic microbes, modulating immune responses, and influencing endocrine and cytokine secretion (Wang et al., 2024; Uzuner et al., 2023). Additionally, advancements in multi-omics technology such as genome sequencing have enabled a more detailed exploration of microbiota’s role in disease. For decades, the 16S rRNA gene has been a fundamental tool in sequence-based bacterial analysis, enabling the identification and classification of diverse microbial communities (Li et al., 2024; Johnson et al., 2019). Its highly conserved regions provide a reliable framework for broad taxonomic comparison, while its variable regions allow for species-level differentiation. This makes 16S rRNA sequencing an essential method in microbial ecology, clinical diagnostics, and microbiome research (Li et al., 2024). These developments can also be used for a deeper understanding of the relationship between microbiota and endometriosis, paving the way for new insights and potential therapeutic strategies.

Among the various theories explaining the origin of endometriosis, the “bacterial contamination hypothesis” posits that bacterial endotoxins contribute to disease progression. Studies have revealed notable contamination of *Escherichia coli* in both menstrual blood and peritoneal fluid in women with endometriosis (Khan et al., 2018). In healthy individuals, the genital microecology maintains a dynamic balance, encompassing normal vaginal anatomy, stable microbiota, a balanced endocrine system, and a functional immune system. However, when dysbiosis disrupts vaginal flora, the protective barrier against pathogens weakens, potentially leading to lower genital infections and serious conditions like cervical precancerous lesions and cancer (Chen et al., 2017; Yu et al., 2024; Hu et al., 2024). Additionally, the gut microbiota plays a crucial role in key biological processes related to endometriosis, including immunity (Stanic et al., 2014), inflammation, estrogen metabolism (Salliss et al., 2022), and even mental status such as depression and anxiety (Davenport et al., 2024). Although the exact mechanisms are still unclear, there is a clear link between gut microbiota (Guo and Zhang, 2024) and endometriosis, underscoring the need for further research to explore this connection and its potential for new therapeutic strategies.

In 2016, Matthias W. et al. proposed a theoretical framework for the involvement of gut microbiota in the pathogenesis of endometriosis (Laschke and Menger, 2016). This hypothesis has gained further support through advanced next-generation sequencing techniques, which have enabled precise identification of microbiota composition in the context of endometrial ectopic lesions. Our systematic review and meta-analysis aim to explore the intricate relationship between the gut microbiome and endometriosis, seeking to identify any consistent patterns or connections across various studies.

## Materials and methods

This study is a rigorous systematic review and meta-analysis, following the PRISMA checklist guidelines (Preferred Reporting Items for Systematic Reviews and Meta-analyses) (Page et al., 2021). Our protocol was registered with PROSPERO in November 2024 (registration number CRD42024611701).

### Literature retrieval strategies

The search strategy was designed by Li with guidance from Professor Wu, covering both English and Chinese electronic databases, including PubMed, MEDLINE, BIOSIS, Cochrane Library, Embase, Wanfang, and CNKI, up to November 20, 2024. Keywords and MeSH terms such as “endometriosis,” “adenomyosis,” “microbiome,” and “gut microbiome” were used in the search. The full search strategy is provided in the Supplementary materials.

### Inclusion criteria

Women over 18 years with a histologically confirmed diagnosis of endometriosis and a control group of women without endometriosis.

Studies focusing on the gut microbiota and comparing individuals with and without endometriosis.

Human observational studies presenting original data.

Use of 16S rRNA or NGS to assess the gut microbiota.

### Exclusion criteria

Excluded publication types: reviews, meta-analyses, consensus statements, conference abstracts, editorials, guidelines, case reports, and case series. Further, in-vitro experiments, intervention trials, animal studies, and studies without a control group. Studies involving women with concurrent inflammatory comorbidities or antibiotic use and non-English language studies were also excluded.

### Material selection, extraction, and quality assessment

During the preliminary data processing stage, the titles and abstracts of all retrieved papers were carefully screened for relevance. Irrelevant papers were excluded, while those with uncertain relevance underwent full-text review. Two researchers (LYY and OYDM) independently assessed eligibility based on predefined inclusion and exclusion criteria, resolving disagreements through discussion with additional reviewers. Data extraction was conducted using a standardized form, capturing key details such as author, publication year, study location, sample characteristics, diagnostic methods, and microbiota indices. Additionally, relevant literature cited in the included studies was reviewed for potential inclusion. A third researcher (LL) was consulted to resolve any disputes. All three researchers had expertise in clinical epidemiology and the relevant domain. The quality of cohort and case–control studies was assessed using the Newcastle-Ottawa Scale (NOS) (Stang, 2010), whereas cross-sectional studies were evaluated with the Agency for Healthcare Research and Quality (AHRQ) checklist (Atkins et al., 2005), this approach aimed to ensure a comprehensive quality assessment across various types of studies. Moreover, if there are any disagreements in quality scoring were discussed within the group and ultimately resolved by consultation with statistical expert Professor Sang from Yunnan University, China.

### Statistical analysis

The data were systematically collected, analyzed, and validated following the established protocols for meta-analysis. The WebPlotDigitizer program was used to extract data from figures. We used RevMan software (version 5.4) to analyses the data by using a 95% confidence interval. Heterogeneity was evaluated using Cochrane’s Q test and I^2^ statistic; a fixed-effect model was used when I^2^ value was below 50%, while a random-effects model was applied otherwise. If needed, a sensitivity analysis was also conducted to evaluate the robustness of the results by using STATA software. Additionally, funnel plots and Egger’s linear regression test were used to assess potential publication bias.

## Results

### Characteristics of studies

**Figure 1:**
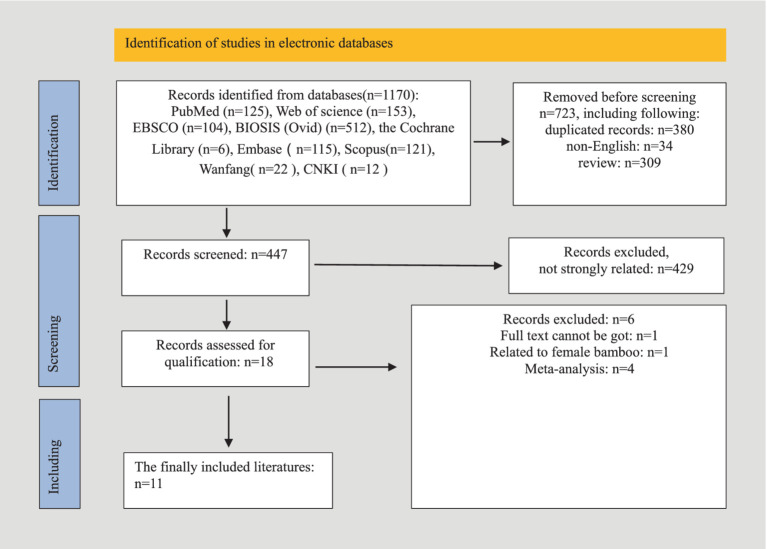
Flowchart for studies included according to PRISMA.

A comprehensive database search yielded 1,170 articles, of which 723 were excluded due to duplication, non-English language, or being review articles. After screening the titles and abstracts of 447 studies, 429 were excluded for lack of relevance. Seventeen studies underwent full-text review, but six were excluded due to unavailable data, non-human focus, or being meta-analyses. Ultimately, 11 studies were included in the systematic review, as detailed in the study selection flowchart (Figure 1). The review included 11 studies, comprising one case–control study and 10 cohort studies, with a total of 1,727 women—433 diagnosed with endometriosis and 1,294 without. Diagnoses were confirmed via laparoscopy, with staging based on r-ASRM criteria (Metzemaekers et al., 2020). One study employed shotgun metagenomics for gut microbiota evaluation, while the others used 16S rRNA sequencing on Illumina or Ion PGM platforms. Detailed study characteristics are summarized in Table 1.

**Table 1:** The summary of the characteristics of all included studies.

Study	Location	Study design	Sample sizes		Diagnosis methods		Sample types	Results
			Trail	Control	Primer	Sequencing		
Ata et al. (2019)	Turkey	Cohort	14	14	V3-V4	16S rRNA	Stool	Stool microbiome predominantly composed of Shigella/Escherichia in 2 women in the stage 3/4 endometriosis group.
Shan et al. (2021)	China	Cohort	12	12	V3-V4	16S rRNA	Feces	The EM group exhibited reduced α diversity in their gut microbiota along with an elevated *Firmicutes/Bacteroidetes* ratio. Significant differences were observed in the abundances of various taxonomic groups, including *Actinobacteria, Tenericutes, Blautia, Bifidobacterium, Dorea,* and *Streptococcus*, between the two groups.
Svensson et al. (2021)	Sweden	Cohort	66	198	V1–V3	16S rRNA	Stool	Controls exhibited higher levels of both alpha and beta diversities compared to patients. At a false discovery rate of q < 0.05, the abundances of 12 bacterial species, belonging to the classes *Bacilli, Bacteroidia, Clostridia, Coriobacteriia*, and *Gamma proteobacteria*, were found to differ significantly between the patient and control groups.
Le et al. (2021)	USA	Cohort	20	9	V4	16S rRNA	Fecal sample	GI bacterial communities were comparable between P-EOSIS and CON subjects who were not taking OCPs, but they differed significantly when OCPs were used.
Huang et al. (2021)	China	Cohort	21	20	V4	16S rRNA	Fecal sample	The fecal microbiota differs significantly between the control group and the EM group. Additionally, the composition of the fecal microbiota varies between patients with early and advanced stages of EM. Furthermore, the depletion of *L. Ruminococcus* in the gut may serve as a potential biomarker for endometriosis.
Pai et al. (2023)	Taiwan	case–control study	37	35	V3-V4	16S rRNA	Fecal samples	There were no significant differences in diversity and composition between individuals with and without Endometriosis in the gut microbiota.
Hicks et al. (2024)	Australia	Cohort	21	43	V3–V4	16S rRNA	Fecal samples	Patients with moderate/severe endometriosis had higher levels of *Fusobacterium*.
Jimenez et al. (2024)	USA	Cohort	35	38	V4	16S rRNA	Rectal swabs	CPP-Endo exhibited an increased abundance of rectal Ruminococcus.
Pérez-Prieto et al. (2024)	Spain	Cohort	136	864	Not available	shotgun metagenomic	Stool samples	No significant differences in diversity were found between women with endometriosis and those without.
Valdés-Bango et al. (2024)	Spain	Cohort	38	46	V3- V4	16S rRNA	Fecal samples	Compared with controls, specific bacterial taxa were identified as either enriched (*Rhodospirillales, Ruminococcus gauvreauii group, Ruminococcaceae,* and *Actinomyces*) or depleted in both the gut and endometrial microbiota of adenomyosis patients.
Do et al. (2024)	USA	cohort	33	15	V4	16S rRNA	Fecal	P-EOSIS exhibited microbial imbalance, marked by the presence of distinct GI/UG bacteria as well as changes in microbial richness and diversity.

### Gut microbiota diversity among various populations

**Figure 2:**
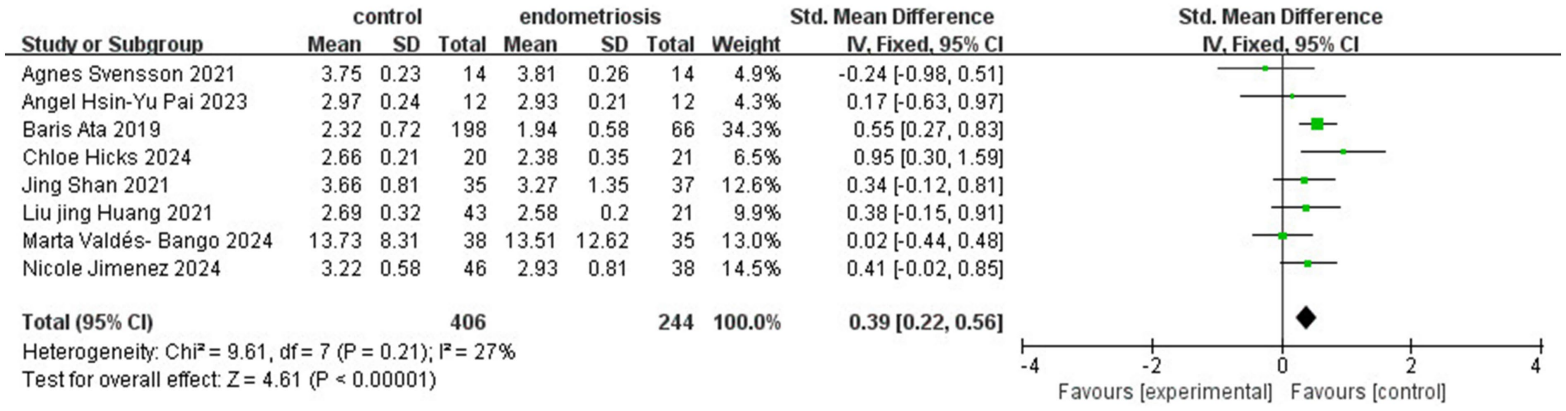
Meta-analysis for Shannon index to evaluate alpha diversity of gut microbiota between women with endometriosis and controls. SD: standard deviation; CI: confidence interval.

**Figure 3:**
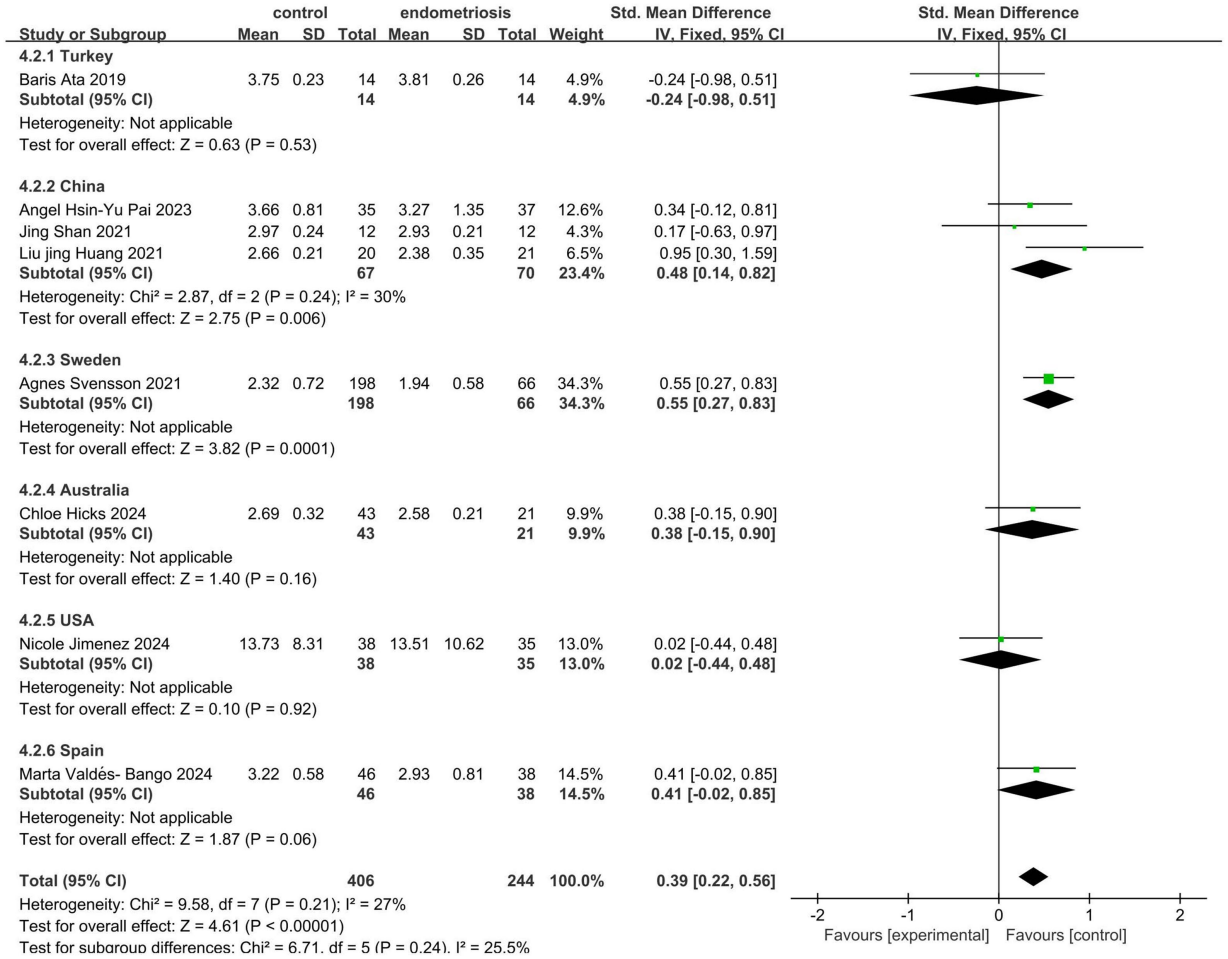
Meta-analysis for Shannon index to evaluate alpha diversity of gut microbiota between subgrouping women with endometriosis and controls. SD: standard deviation; CI: confidence interval.

The analysis of gut microbiota diversity revealed significant differences in alpha diversity between women with endometriosis and control groups. Using the Shannon Index, a notable difference was observed (SMD = 0.39; 95% CI = 0.22 to 0.56; *p* < 0.00001; *I*^2^ = 27%) across eight studies involving 650 participants (Figure 2). Subgroup analysis by ethnicity showed significant differences for Chinese (SMD = 0.48; 95% CI = 0.14 to 0.82; *p* = 0.006; *I*^2^ = 30%), Swedish, (SMD = 0.55; 95% CI = 0.27 to 0.83; *p* = 0.0001; *I*^2^ = 30%) and Spanish (SMD = 0.34; 95% CI = −0.02 to 0.85; *p* < 0.06; *I*^2^ = 27%), highlighting the correlation between gut microbiota diversity and endometriosis across different demographic groups (Figure 3).

**Figure 4:**
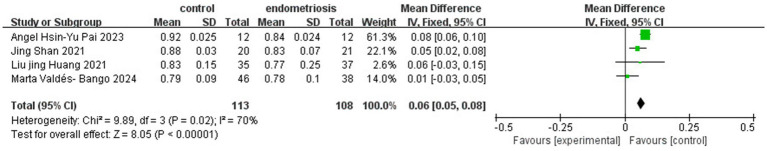
Meta-analysis for Simpson index to evaluate alpha diversity of gut microbiota between women with endometriosis and controls. SD: standard deviation; CI: confidence interval.

**Figure 5:**
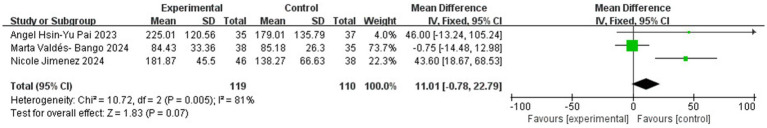
Meta-analysis for gut microbiota alpha diversity assessed by Chao index between women with endometriosis and controls. SD: standard deviation; CI: confidence interval.

Further assessment using the Simpson Index demonstrated significant differences in alpha diversity richness between the endometriosis and control groups (SMD = 0.91; 95% CI = 0.08 to 1.73; *p* = 0.03; *I*^2^ = 87%) across four studies with 221 participants (Figure 4). However, the Chao Index did not reveal any significant differences in gut microbiota richness (SMD = 0.37; 95% CI = −0.09 to 0.82; *p* = 0.11; *I*^2^ = 66%) based on data from three studies involving 219 participants (Figure 5). These results suggest that while specific alpha diversity measures indicate significant variations, others remain inconclusive.

Beta diversity analysis, conducted in 10 of the 11 studies, showed clear distinctions in the gut microbiota composition between the groups. Seven studies employed Principal Coordinate Analysis (PCoA), two used the Bray–Curtis dissimilarity index, and one applied Principal Component Analysis (PCA). However, due to variability in reporting formats, a quantitative comparison of microbiota compositions was not feasible, and detailed descriptions are provided in Tables 2, 3.

**Table 2:** Microbiota diversity analysis of included studies.

Study	Alpha diversity		Beta diversity		Composition
Ata et al. (2019)	Shannon index	Control: 3.75 ± 0.23; endometriosis: 3.81 ± 0.26	PCoA	*p* = 0.635	The composition of the gut microbiota was comparable between the endometriosis group and the control group.
Shan et al. (2021)	Sob	Control:167 ± 53.6 Endometriosis:162.06 ± 51.75	PCoA	*R* = 0.2616, *p* = 0.001	the gut microbiota of the EM group exhibited reduced α diversity and an increased *Firmicutes*/*Bacteroidetes* ratio compared to the control group. Furthermore, there were significant differences in the abundances of several taxa, including *Actinobacteria, Tenericutes, Blautia, Bifidobacterium, Dorea*, and *Streptococcus*, between the two groups.
	Ace	Control:189.65 ± 58.38 Endometriosis:198.47 ± 43.36			
	Shannon index	Control: 2.97 ± 0.24 Endometriosis: 2.93 ± 0.21			
	Simpson index	Control: 0.92 ± 0.025 Endometriosis: 0.84 ± 0.024			
Svensson et al. (2021)	Shannon index	Control:2.32 ± 0.72 Endometriosis:1.94 ± 0.58	Bray–Curtis dissimilarity index	–	The abundances of 12 bacterial species, belonging to the classes Bacilli, Bacteroidia, Clostridia, Coriobacteriia, and Gamma proteobacteria, were significantly different between patients and controls.
Le et al. (2021)	Simpson’s index	Details not given	PCoA	–	GI bacterial communities were comparable between P-EOSIS and CON subjects who were not taking OCPs, but they differed significantly when OCPs were used.
	Simpson’s evenness	Details not given			
Huang et al. (2021)	Shannon index	Control: 2.66 ± 0.21 Endometriosis: 2.38 ± 0.35	PCoA	The variations in microbiome composition between the two groups are evident along the PCoA1 axis.	Endometriosis patients exhibit distinct microbial communities compared to the control group, particularly in feces, where the genus *Ruminococcus* has been identified as a potential gut biomarker.
	Simpson’s index	Control: 0.88 ± 0.03 Endometriosis: 0.83 ± 0.07			
Pai et al. (2023)	Shannon index	Control: 3.66 ± 0.81 endometriosis: 3.27 ± 1.35	PCoA	The gut microbiota did not show clustering based on the presence or absence of the disease.	The gut microbiota of the endometriosis group showed statistically significant enrichment in specific taxa, namely bacteria belonging to the *Erysipelotrichia* class (p = 0.0286), *Erysipelotrichales* order (p = 0.0286), *Erysipelotrichaceae* family (*p* = 0.0286), as well as the *Eisenbergiella* genus (*p* = 0.0474) and *Hungatella* genus (*p* = 0.0497).
	Simpson index	Control: 0.83 ± 0.15 endometriosis: 0.77 ± 0.25			
	Chao	Control: 225.01 ± 120.56 endometriosis: 179.01 ± 135.79			
	Good’s coverage	Control: 0.63 ± 0.21 endometriosis: 0.70 ± 0.20			
Hicks et al. (2024)	Shannon index	Control: 2.69 ± 0.32 endometriosis: 2.58 ± 0.21	PCoA	There was significant differences observed.	Lactobacillus was more prevalent in ENDO stool microbiota samples.
Jimenez et al. (2024)	Shannon index	Control: 13.73 ± 8.31 endometriosis: 13.51 ± 10.62	PCA		
	Chao	Control: 84.43 ± 33.36 endometriosis: 85.18 ± 26.30			
Valdés-Bango et al. (2024)	Chao	Control:181.87 ± 45.50 endometriosis: 138.27 ± 66.63	Bray–Curtis dissimilarity index	Albeit not statistically significant	Fecal samples from the adenomyosis group showed a significant increase in the abundance of the *Rhodospirillales* order and the *Ruminococcus gauvreauii* group genus.
	Shannon index	Control: 3.22 ± 0.58 endometriosis: 2.93 ± 0.81			
	Simpson index	control: 0.79 ± 0.09 endometriosis: 0.78 ± 0.10			
Do et al. (2024)	Faith’s PD	Subgrouping	PCoA	Bacterial composition in fecal samples from control patients varied significantly.	

**Table 3:** Compared with the control, special findings for microbiota compositions in endometriosis(legend:↑ = increased,↓ = decreased).

Study	Phylum	Class	Order	Family	Genus	Species
Ata et al. (2019)	–	–	–	–	*Sneathia↓,* *Barnesella↓, Gardnerella↓*	–
Shan et al. (2021)	*Firmicutes/Bacteroidetes*↑	–	–	–	*Bifidobacterium↓, Blautia↓, Dorea↓, Streptococcus↓, [Eubacterium] hallii↓* *Lachnospira↑, [Eubacterium] eligens_group↑*	–
Svensson et al. (2021)	–	*Clostridia↑* *Bacteroidia↓* *Alphaproteobacteria↓* *Mollicutes↓*	–	–	*Lachnospira↓*	–
Le et al. (2021)	*Firmicute/Bacteroidetes↑*	–	–	–	–	–
Huang et al. (2021)	–	–	–	–	–	*Lachnospiraceae Ruminococcus↑*
Pai et al. (2023)	–	*Erysipelotrichia↑*	*Erysipelotrichia↑*	*Erysipelotrichaceae↑* *Micrococcaceae↑*	*Eisenbergiella* *Hungatella↑*	–
Hicks et al. (2024)	–	–	–	*veillonellaceae↑* *Lactobacillaceae↑*	*Lactobacillus↑* *Phascolartobacterium↑*	–
Valdés-Bango et al. (2024)	–	–	*Rhodospirillales↑,*	–	*Ruminococcus gauvreauii↑*	*Rhodospirillales↑, Ruminococcus↑, gauvreauii group↑, Ruminococcaceae↑, Actinomyces↑*
Do et al. (2024)	–	–	–	–	*Finegoldia, Bacteroides, Corynebacterium, Anaerococcus, eptoniphilus, Lactobacillus, Blautia, Prevotella, aecalibacterium, Ruminococcus*	*–*

### Publication bias analysis

**Figure 6:**
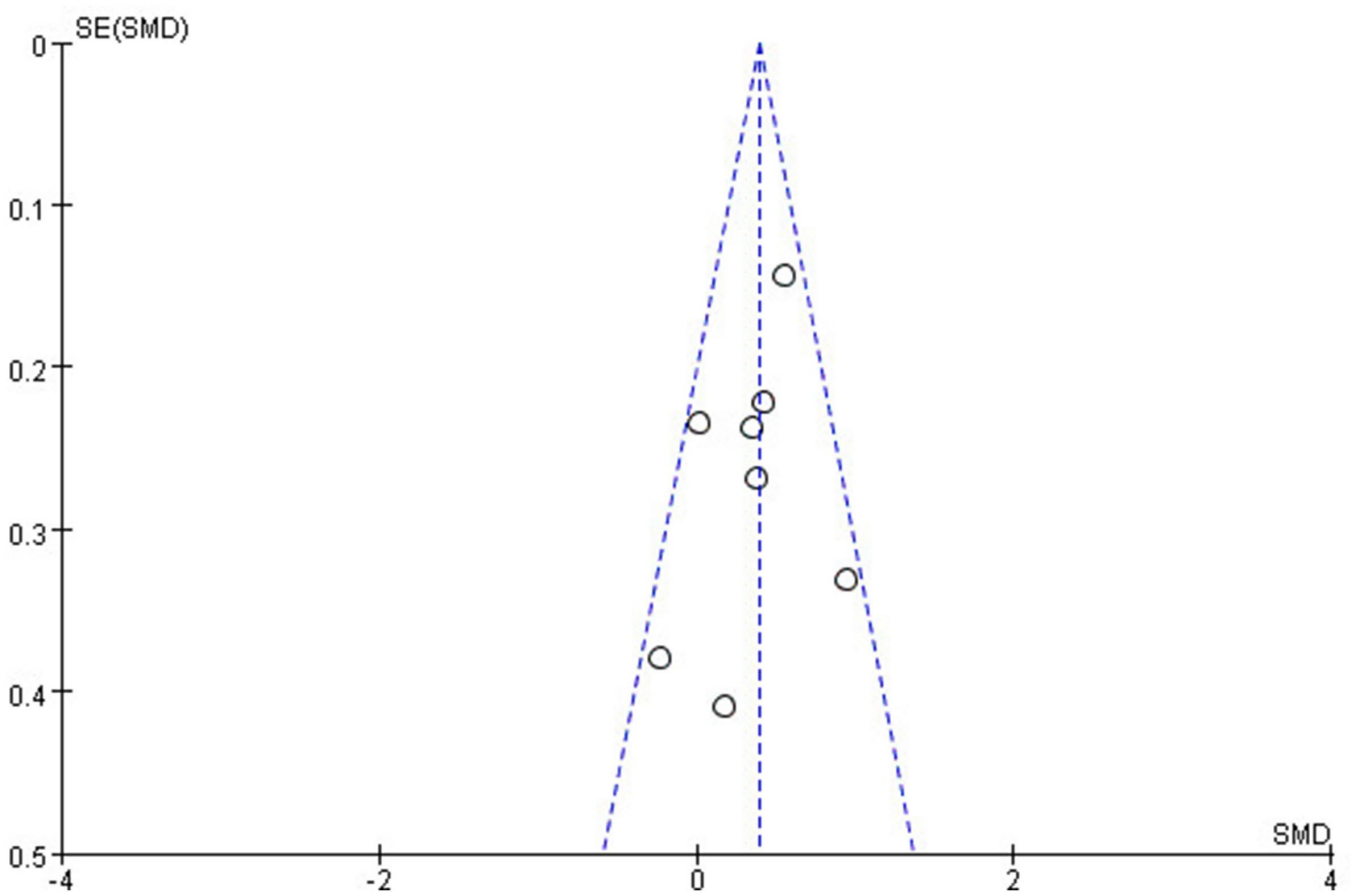
Funnel plot from the Shannon index that was used most frequently in all the studies.

The methodological quality of the included studies was assessed using the Newcastle-Ottawa Scale (NOS), with most studies scoring seven stars or higher, indicating high quality and low risk of bias. A funnel plot for the Shannon Index suggested symmetry, indicating no publication bias in this meta-analysis (Figure 6). Moreover, we have also conducted Egger’s linear regression test to quantitatively assess publication bias in the included studies by using STATA to ensure statistical accuracy and reproducibility. The results showed that *p* = 0.3073, indicating no publication bias. These findings underscore the importance of gut microbiota diversity in endometriosis research while highlighting the need for standardization in reporting methods to facilitate future meta-analyses (Table 4).

**Table 4:** The quality of the included studies assessed by using the Newcastle-Ottawa scale.

Study	Selection				Comparability	Exposure result			Total
	1	2	3	4	1	1	2	3	9
Case control									
Pai et al. (2023)	*	*	*	*	*	*	–	–	7
Cohort									
Ata et al. (2019)	–	*	*	*	*	*	*	–	7
Shan et al. (2021)	*	*	*	*	*	*	–	–	7
Svensson et al. (2021)	*	*	*	*	*	*	–	–	7
Le et al. (2021)	*	*	*	*	*	*	–	–	7
Huang et al. (2021)	*	*	*	*	*	*	–	–	7
Hicks et al. (2024)	*	*	*	*	*	*	–	–	7
Jimenez et al. (2024)	*	*	*	*	*	*	–	–	7
Pérez-Prieto et al. (2024)	*	*	*	*	*	*	–	–	7
Valdés-Bango et al. (2024)	*	*	*	*	*	*	–	–	7
Do et al. (2024)	*	*	*	*	*	*	–	–	7

Case–control studies: selection: (1) is the case definition adequate? (2) Representativeness of cases; (3) selection of controls; (4) definition of controls; comparability: (1) comparability of cases and controls based on design or analysis; exposure: (1) exposure verification; (2) same calculation method for cases and controls; (3) Nonresponse rate. Cohort study: selection: (1) representativeness of the exposed cohort; (2) selection of the unexposed cohort; (3) exposure verification; (4) demonstration that the result of interest was not present at the beginning of the study; comparability: (1) comparability of cohorts based on design or analysis; result: (1) result evaluation; (2) follow-up was long enough for results to occur; (3) Adequacy of cohort follow-up.

### Sensitivity analysis

**Figure 7:**
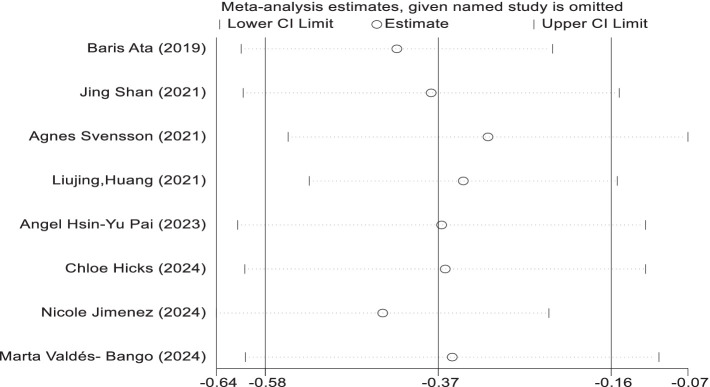
Sensitive analysis of included studies.

In the sensitivity analyses, while analysis using the random effect model showed that no trials had substantial influence on the pooled analysis. The results are similar in the two models (Figure 7).

## Discussion

The increasing prevalence of endometriosis is placing a significant burden on public health, with symptoms such as abdominal masses, pelvic pain, infertility, and psychological distress. Unfortunately, the limited understanding of the disease’s underlying causes restricts treatment options to medication and surgery, unlike preventable diseases such as cervical cancer. Over billions of years, diverse microbial ecosystems have evolved in humans, fostering a mutually beneficial relationship between humans and their resident bacteria, particularly in the lower gastrointestinal tract (Ley et al., 2006; Ley et al., 2008; Van Hul et al., 2024). A healthy gut is characterized by a balanced microbial composition, and dysbiosis indicates a disruption in bodily function (Van Hul et al., 2024). Researchers have proposed that gut microbiota play a key role in the development of endometriosis (Qin et al., 2022; Talwar et al., 2022; Jiang et al., 2021; Kovács et al., 2021). The “bacterial contamination hypothesis” introduced in Japan emphasizes the strong connection between microbiota and endometriosis (Khan et al., 2018). In this systematic review and meta-analysis, we aimed to explore the relationship between gut microbiota and endometriosis to gain a deeper understanding of the disease’s mechanisms.

In our systematic review and meta-analysis, we observed significant differences in alpha diversity between women with endometriosis and the control group. Several indices, including Shannon, Chao, Simpson, Simpson evenness, Good’s coverage, ACE, and Sob, were used to assess alpha diversity. Of these, the Shannon, Simpson, and Chao indices were the most frequently employed. The Shannon index evaluates species diversity, considering both abundance and distribution, where higher values indicate greater diversity. Simpson’s index measures the relative abundance of species, with higher scores reflecting greater community diversity. The Chao index estimates the total number of operational taxonomic units (OTUs), often used to assess species richness. Our meta-analysis revealed gut microbiota dysbiosis in women with endometriosis, with higher alpha diversity observed in the control group. As reported in previous studies, imbalances in the gut microbiome disrupt immune function, triggering inflammatory responses that elevate pro-inflammatory cytokines, impair immune surveillance, and alter immune cell profiles (Visconti et al., 2019). These disruptions lead to chronic inflammation, fostering an environment conducive to cellular adhesion, angiogenesis, and fibrosis, which can perpetuate the progression of endometriosis, contributing to its chronicity and severity (Fan et al., 2023). Another review article published by Iavarone et al. identified significant microbial shifts at the genus level, with increased *Prevotella, Blautia,* and *Bifidobacterium* and decreased *Paraprevotella*, *Ruminococcus*, and *Lachnospira*, as well as notable changes in *Proteobacteria* abundance following abdominal hysterectomy (Iavarone et al., 2023). Our meta-analysis further reinforces these findings by demonstrating significant alpha diversity differences between endometriosis and control groups, particularly through Shannon (SMD = 0.39; *p* < 0.00001) and Simpson (SMD = 0.91; *p* = 0.03) indices, while the Chao Index did not reveal significant differences (SMD = 0.37; *p* = 0.11).

Studies by Shan et al. (2021) and Le et al. (2021) revealed an accumulation of the *Firmicutes*/*Bacteroidetes* phylum in females with endometriosis, while Svensson et al. (2021) identified an increase in the *Clostridia* class, belongs to the *Firmicutes* phylum. These bacteria are capable of producing short-chain fatty acids (SCFAs), which may contribute to the onset of endometriosis (Highlander et al., 2012). Additionally, an imbalance between the *Firmicutes* and *Bacteroidetes* phyla has been observed in women with endometriosis, a disruption that may play a significant role in the dysregulation of estrogen metabolism. This imbalance is particularly important because certain bacteria in these phyla possess genes encoding glucuronidase, an enzyme involved in estrogen metabolism. Altered glucuronidase activity can lead to changes in estrogen levels, which in turn may stimulate the growth and spread of endometrial cells outside the uterine cavity, contributing to the development and progression of endometriosis (Kovács et al., 2021; Highlander et al., 2012; Gloux et al., 2010).

Our systematic review found a reduction in the abundance of bacteria from the genera *Bifidobacterium* (Shan et al., 2021), and *Lachnospira* (Svensson et al., 2021), which are known for their ability to adhere to intestinal mucosal cells, forming a protective barrier against pathogenic bacteria. This barrier is essential for maintaining gut health and preventing disease. In a similar vein, Leonardi et al. conducted a systematic review 4 years ago on the relationship between the microbiome and endometriosis, including both human and animal studies. They observed an increased presence of *Proteobacteria, Enterobacteriaceae, Streptococcus* spp., and *Escherichia coli* in individuals with endometriosis, along with an increase in *Gardnerella* and the phylum *Firmicutes* (Leonardi et al., 2020). Their findings align with ours, though our review focused exclusively on human studies.

Human gut microbiota, consisting of 100 trillion cells shaped by evolutionary selection pressures (Ley et al., 2006), is influenced by various factors such as gender, age, BMI, diet, lifestyle, and antibiotic use (Li et al., 2024). In the context of endometriosis, specific strains of *Lactobacillus* and *Bifidobacterium* show promise in reducing inflammation (Itoh et al., 2010). These probiotics strengthen the intestinal barrier, preventing endotoxins from entering the bloodstream and reducing systemic inflammation. Additionally, they aid in estrogen metabolism through the gut-liver axis, potentially lowering excess estrogen that exacerbates the disease. Probiotics also have the potential to enhance regulatory T cell activity, which could alleviate the immune dysregulation associated with endometriosis. Although clinical trials on probiotics for endometriosis are limited, preliminary animal studies and small human trials suggest benefits in reducing pain and inflammation. Interventions such as probiotics, prebiotics, synbiotics, fecal microbiota transplantation, and dietary changes show promise in managing endometriosis by regulating inflammation, immune responses, and estrogen metabolism. Future clinical trials will be vital for evaluating the safety and effectiveness of these microbiota-targeted therapies.

This research is a rigorous meta-analysis that synthesizes data from 11 studies involving 1,727 women, including 433 diagnosed with endometriosis and 1,294 controls, providing a comprehensive evaluation of the relationship between gut microbiota and endometriosis. The large sample size enhances the reliability of our findings, while the use of advanced statistical methods, including Egger’s test for publication bias, sensitivity analysis, and heterogeneity assessment, ensures the validity and robustness of the results. Additionally, our study is grounded in the most up-to-date research, contributing to a timely and relevant understanding of the microbiome’s role in endometriosis. These strengths collectively support the significance of our study in advancing knowledge in the field.

Several limitations in our meta-analysis should be considered. First, the lack of uniformity in sequencing methods, with some studies using 16S rRNA sequencing and others employing shotgun metagenomic sequencing, hindered our ability to effectively compare results, as these methods may yield different levels of detail and accuracy. Additionally, the absence of a standardized index for assessing both alpha and beta diversity made it challenging to draw consistent conclusions about gut microbiota composition and its relationship to the studied outcomes. Second, we observed significant heterogeneity in the data, likely due to differences in study populations, sample sizes, and experimental designs, complicating data pooling and interpretation. Furthermore, the relatively small number of included studies may have affected the robustness and generalizability of our findings, and there is a risk of publication bias, with positive studies potentially being overrepresented. Additionally, the inclusion of only English-language studies may represent another limitation, as it could introduce language bias and exclude relevant research published in other languages. These limitations should be kept in mind when interpreting the results, and future research should address these issues to draw more definitive conclusions.

## Conclusion

Our meta-analysis identified significant differences in gut microbiota diversity between women with endometriosis and controls, as assessed by the Shannon and Simpson indices. Subgroup analysis confirmed these differences among various countries’ populations including Chinese, Swedish, and Spanish populations compared to others. Beta diversity analysis also revealed distinct microbiota composition, though variability in reporting formats hindered quantitative comparisons. These findings emphasize the role of gut microbiota in endometriosis and highlight the need for standardized reporting methods in future studies. While no definitive microbiota composition predictor was found, a potential association with endometriosis warrants further investigation. Advances in high-resolution sequencing and multi-omics offer promising avenues for discovering new pathways, which may lead to innovative diagnostic and therapeutic strategies.

## Data Availability

The original contributions presented in the study are included in the article/supplementary material, further inquiries can be directed to the corresponding author/s.
